# Nonideal nest box selection by tree swallows breeding in farmlands: Evidence for an ecological trap?

**DOI:** 10.1002/ece3.8323

**Published:** 2021-11-09

**Authors:** Ève Courtois, Dany Garant, Fanie Pelletier, Marc Bélisle

**Affiliations:** ^1^ Département de Biologie Université de Sherbrooke Sherbrooke Quebec Canada

**Keywords:** aerial insectivore, ecological trap, habitat preference, habitat quality, house sparrow, settlement cue

## Abstract

Animals are expected to select a breeding habitat using cues that should reflect, directly or not, the fitness outcome of the different habitat options. However, human‐induced environmental changes can alter the relationships between habitat characteristics and their fitness consequences, leading to maladaptive habitat choices. The most severe case of such nonideal habitat selection is the ecological trap, which occurs when individuals prefer to settle in poor‐quality habitats while better ones are available. Here, we studied the adaptiveness of nest box selection in a tree swallow (*Tachycineta bicolor*) population breeding over a 10‐year period in a network of 400 nest boxes distributed along a gradient of agricultural intensification in southern Québec, Canada. We first examined the effects of multiple environmental and social habitat characteristics on nest box preference to identify potential settlement cues. We then assessed the links between those cues and habitat quality as defined by the reproductive performance of individuals that settled early or late in nest boxes. We found that tree swallows preferred nesting in open habitats with high cover of perennial forage crops, high spring insect biomass, and high density of house sparrows (*Passer domesticus*), their main competitors for nest sites. They also preferred nesting where the density of breeders and their mean number of fledglings during the previous year were high. However, we detected mismatches between preference and habitat quality for several environmental variables. The density of competitors and conspecific social information showed severe mismatches, as their relationships to preference and breeding success went in opposite direction under certain circumstances. Spring food availability and agricultural landscape context, while related to preferences, were not related to breeding success. Overall, our study emphasizes the complexity of habitat selection behavior and provides evidence that multiple mechanisms may potentially lead to an ecological trap in farmlands.

## Graphical abstract

We studied the adaptiveness of nest box selection in a tree swallow population breeding over a 10‐year period in a network of 400 nest boxes distributed along a gradient of agricultural intensification in southern Québec, Canada. We first examined the effects of multiple environmental and social habitat characteristics on nest box preference to identify potential settlement cues, then assessed the links between those cues and habitat quality as defined by the reproductive performance of individuals that settled early or late in nest boxes. We found multiple mismatches potentially indicating ecological trap mechanisms.

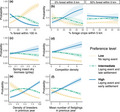

## 1 INTRODUCTION

Breeding habitat selection decisions influence fitness of animals through the costs and benefits of habitat use (Hildén, 1965; Martin, 1998; Morris, 2003). Natural selection should thus favor the evolution of adaptive behavioral responses whereby individuals preferentially use habitats that maximize their fitness (Fretwell & Lucas, 1970; Hale & Swearer, 2016). Because animals cannot always evaluate the quality of habitats in terms of fitness returns due to various constraints such as time and energy, they often rely on cues that reflect, directly or indirectly, the expected fitness outcome of different habitat options (Robertson & Hutto, 2006; Stamps, 2001; Stamps & Krishnan, 2005). Those cues include habitat characteristics perceived through personal observation (e.g., landscape features, Bollinger, 1995; Hollander et al., 2011; food availability, Burke & Nol, 1998) or associated with personal performance in a given environment (Lagrange et al., 2017; Switzer, 1997), or the behavior or performance of other individuals of the same or different species (Doligez, 2002; Mönkkönen et al., 1999; Pärt et al., 2011).

Social information likely integrates the effect of many environmental factors on expected breeding success via their effects on the distribution and performance of conspecifics and heterospecifics (Doligez et al., 2003). Social information can be gathered by prospecting behavior during or at the end of a breeding event, so to be used in future reproduction, and is thought to be a reliable settlement cue if habitat quality is sufficiently predictable (Boulinier & Danchin, 1997; Doligez et al., 2003; Valone & Templeton, 2002).

Ecological traps arise from mismatches between the preferences for some habitat characteristics and the fitness outcome of such preferences, and occur when poor‐quality habitats are preferred although better ones are available (Battin, 2004; Pärt et al., 2007; Schlaepfer et al., 2002). Rapid environmental changes, notably human‐induced ones, can amplify such mismatches between expected and realized fitness (Robertson & Hutto, 2006; Schlaepfer et al., 2002). Ecological traps can result from various mechanisms affecting either the attractiveness of habitats, their actual quality, or both (Robertson et al., 2013). The consequences of ecological traps on population dynamics depend on the severity of the trap, which in turn varies according to the proportion of poor‐quality habitats, their relative attractiveness, and the magnitude of their fitness costs (Delibes et al., 2001; Hale et al., 2015). Maladaptive habitat selection is thus a continuum, with most severe cases being attractive sink habitats that could drive population decline and extirpation (Battin, 2004; Delibes et al., 2001; Pärt et al., 2007).

Among anthropogenically perturbed ecosystems susceptible to creating ecological traps, farmlands have received much attention (Hale & Swearer, 2016). Because human activities on farmlands are numerous and diversified, as well as temporally unpredictable (e.g., crop rotation, soil preparation, harvest, agrochemicals inputs, livestock grazing), many different mechanisms have been found to trap various taxa into making bad habitat choices (e.g., traps caused by machinery: Bollinger et al., 1990; Touihri et al., 2019; by pesticides: Duchet et al., 2018; Takahashi, 2007; Gervais et al., 2003; or by changes in landscape structure: Rodenhouse & Best, 1983; Morris & Gilroy, 2008).

Here, we studied the adaptiveness of nest box selection within a tree swallow (*Tachycineta bicolor*) population breeding along a gradient of agricultural intensification in southern Québec, Canada. We hypothesized that environmental cues, such as landscape context and spring food availability, lead to nonideal breeding habitat selection, while both hetero‐ and conspecific social information provide more integrative and thus reliable cues of habitat quality for tree swallows breeding in agroecosystems (see Table 1 for rationale and references). For instance, farmland birds face numerous stubble fields upon spring arrival. These stubble fields may transform into a variety of vegetation covers depending on the crops sown. Moreover, crops vary strongly in their susceptibility to pests and can thus be subjected to different systematic and reactive pesticide treatments. Food availability may hence vary unpredictably through space and time, and spring food availability may thus not correlate well with food availability during provisioning.

**TABLE 1:** Justification of the explanatory variables used to assess the determinants of habitat preference and their impact on the reproductive success of tree swallows in a nest box network in southern Québec, Canada, between 2009 and 2018

Explanatory variable	Type	Abbreviation	Justification
% Forest within 100 m of nest box	Landscape	Forest 100 m	Tree swallows settle first in nest sites far from forest edges to avoid interspecific competition and nest predation and to maximize flight area (Rendell & Robertson, 1990)
% Forage crops within 5 km of nest box	Landscape	Forage crops 5 km	Nest box occupancy decreases with intensive cultures, while both number of fledglings and fledging probability increase with forage crops (hay, alfalfa, and clover), pastures and natural grasslands (Ghilain & Bélisle, 2008)
Interaction: % Forest within 5 km × % Forage crops within 5 km of nest box	Landscape	Forest 5 km × Forage crops 5 km	Relative use and suitability of an open habitat may depend on its amount and on the amount and suitability of alternative habitats, and how they are arranged in space as this affects functional connectivity (Bruun & Smith, 2003; Mysterud & Ims, 1998; Sutherland, 1996)
% Water + wetlands within 10 km of nest box	Landscape	Water 10 km	Tree swallows are known to breed near water and wetlands over which they forage for insects of better nutritional quality (Bellavance et al., 2018; Twining et al., 2016, 2018; Winkler et al., 2011). Agricultural intensification in southern Québec reduced wetlands (Bélanger & Grenier, 2002; Benton et al., 2003; Jobin et al., 2003), and strongly contaminated surface waters with pesticides (Giroux, 2019; Montiel‐León et al., 2019), which may negatively affect swallows either directly through toxic effects or indirectly by reducing the availability of aquatic insects (Gibbons et al., 2015; Hallmann et al., 2014; Morrissey et al., 2015)
Mean spring insect dry biomass on farm (g/day)	Food	Insects	Migrant aerial insectivores may be constrained to assess the quality of habitats based on the information available upon their arrival on breeding grounds, yet insect prey availability and quality can be modulated by an unpredictable use of pesticides in both space and time (Botías et al., 2019; Mulé et al., 2017; Pisa et al., 2015; Rioux Paquette et al., 2013). Prey availability is typically positively correlated to multiple reproductive success components in tree swallows (McCarty & Winkler, 1999; Nooker et al., 2005)
Density of house sparrows on farm (no. nest boxes occupied)	Heterospecific social information	Competitor density	Tree swallow occupancy is negatively associated with house sparrow abundance, likely because of competition for nesting sites (Robillard et al., 2013)
Density of tree swallows on farm (no. of nest boxes occupied) in the previous year	Conspecific social information	Density @ *t*−1	Cues associated with the location and breeding performance of conspecifics may act as reliable information integrating multiple environmental effects on breeding success (Boulinier & Danchin, 1997; Valone & Templeton, 2002). Conspecific aggregations may thus be attractive, inasmuch as they could also provide breeding benefits (Lagrange et al., 2017; Lombardo, 1987; Pegan et al., 2018)
Mean no. of fledglings on farm in the previous year	Conspecific social information	Success @ *t*−1	Individuals may use previous year breeding success cues at the farm or nest box level to guide settlement decisions (Lagrange et al., 2017)
Mean spring temperature on farm (°C)	Control		Higher spring temperatures are associated with earlier timing of breeding (Bourret et al., 2015; Dunn & Winkler, 1999)
Mean spring precipitations on farm (mm)	Control		Higher precipitations reduce insect and bird activity (Cox et al., 2019; Grüebler et al., 2008; Robbins, 1981)
Longitude of nest box	Control		Tree swallows settle earlier in the western part of the study system, possibly because they use the St. Lawrence River and some of its main tributaries as migratory routes (Porlier et al., 2009)
Latitude of nest box	Control		Tree swallows arrive in the study system by the south, and breeding phenology is known to depend on latitude in our study system (Bourret et al., 2015)

Our approach followed the two‐step model suggested by Pärt et al. (2007) to study ecological traps by focusing on individual selection decisions in order to identify potential mechanisms of nonideal habitat selection. First, we identified potential cues used by tree swallows for nest box selection by determining which environmental and social habitat characteristics were associated with preference as determined by nest box occupancy and settlement date. Second, we tested whether these cues were good predictors of habitat quality as defined by two components of reproductive success: (1) number of hatchlings and (2) fledging success. We further analyzed the relationship between habitat characteristics and reproductive success separately for early and late settlers, who differ in their age structure, body condition, and breeding phenology (Lozano et al., 1996; Møller, 1994; Porlier et al., 2009), in order to assess whether they experience different constraints that would translate into differential habitat selection adaptiveness.

## 2 METHODS

### 2.1 Species and study area

Tree swallows are small migratory passerines that feed on insects in flight. They breed all over North America but, as many other aerial insectivores, have been declining over large portions of their breeding range, especially in the north‐eastern parts (Michel et al., 2016; Nebel et al., 2010; Shutler et al., 2012). As an obligate secondary cavity nester, this semi‐colonial species readily uses nest boxes.

**FIGURE 1:**
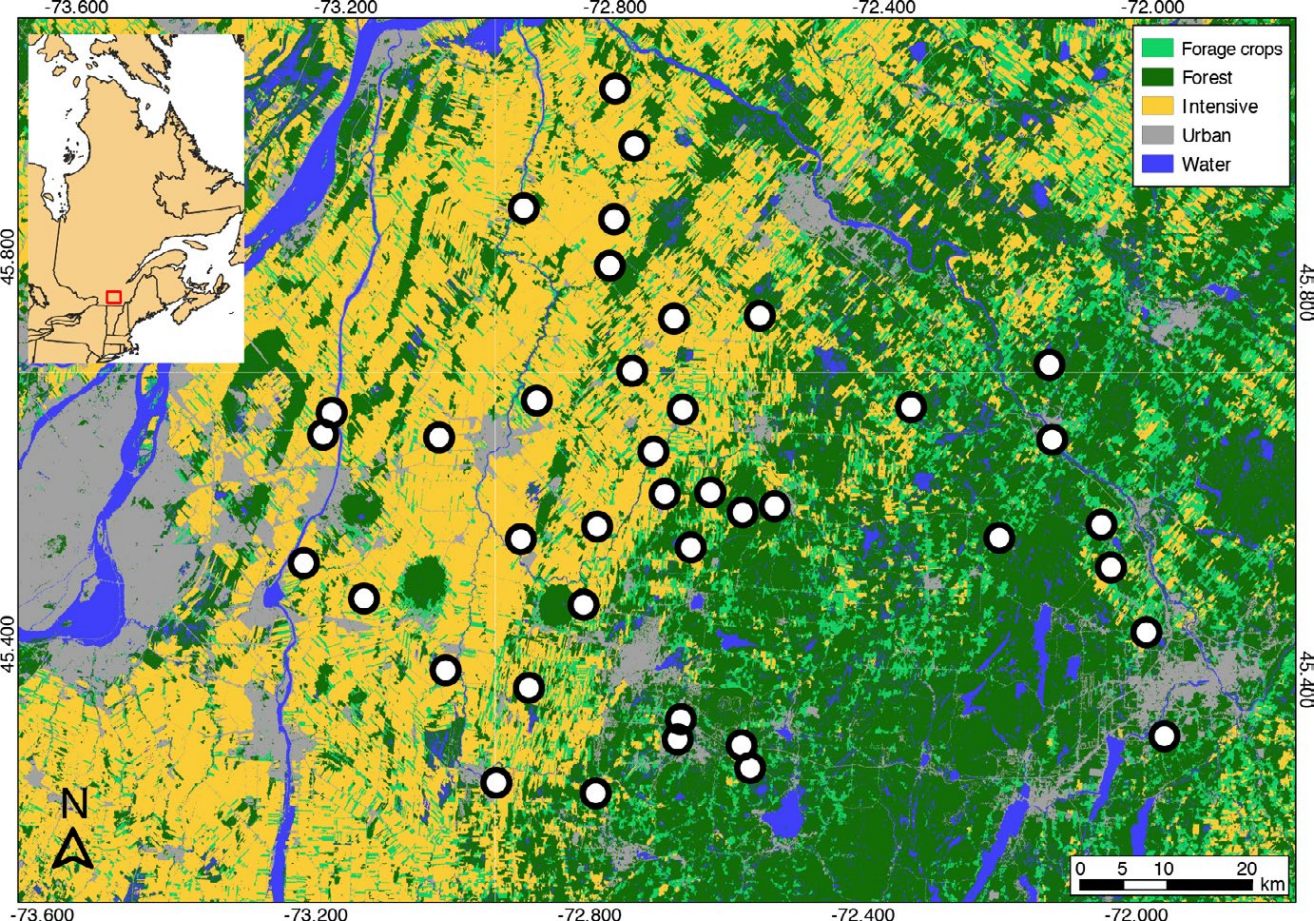
Distribution of the 40 farms in southern Québec, Canada, where nest box preference and breeding ecology of tree swallows were monitored between 2009 and 2018 along a gradient of agricultural intensification. Land cover types are based on a mosaic of classified satellite images (Agriculture & Agri‐Food Canada, 2018). The study system is characterized by a gradient of intensive agriculture in the West (yellow), which shifts to a less intensive and more forested landscape in the East (light and dark green). Each farm is represented by a circle

The study area included 40 farms distributed along a gradient of agricultural intensification covering approximately 10,200 km^2^ in southern Québec, Canada (Figure 1). Three land cover types dominated the study system: low‐intensity agricultural fields (i.e., hay, alfalfa (*Medicago sativa*), clover (*Trifolium* spp.), and pastures, henceforth referred to as “forage crops”); intensive agricultural fields (i.e., annual row crops mainly composed of corn (*Zea mays*), soybean (*Glycine max*), and wheat (*Triticum* spp.)); and forest. While the eastern part of the system was dominated by forest and forage crops, the western part was largely dominated by intensive crops. Pairwise correlations between % forage crops, % intensive crops, and forest were thus high (5‐km scale over all years: *r*_forage,intensive_ = −.78, *r*_forest,intensive_ = −.95, and *r*_forage,forest_ = .66). By considering the relative covers of forage crops and forest, we hence automatically captured information about intensive crops in our analyses (see below).

Each farm included 10 identical nest boxes mostly arranged in a row along field margins and separated by at least 50 m to limit intra‐ and interspecific competition (see Ghilain & Bélisle, 2008 for further details on the study system). A Thermochron iButton device was fixed on the outside of one nest box on each farm to record hourly ambient temperature (model DS1922L; Embedded Data Systems). A pluviometer collected precipitation data (millimeters of rainfall) on each farm. We used the mean daily temperature, which was correlated with maximum and minimum daily temperature (*r* = .89 and *r* = .66, respectively), and mean daily rainfall between May 1 and May 15 to characterize spring climate. This time window was chosen to represent the period during which swallows are actively selecting nesting sites; 95% of nest boxes that hosted a laying event contained nesting material by May 15, meaning that habitat selection mainly occurs before this date, while 90% of laying events occurred after May 15.

### 2.2 Nest monitoring

We monitored nest boxes every other day from 2009 to 2018 starting in the first week of May. We recorded the occurrence of nest materials, the laying date (first egg), and the number of eggs, hatchlings, and fledglings. Only the first breeding attempts of each box were kept for the analyses because second clutches are uncommon (11% of all tree swallows’ breeding attempts between 2009 and 2018) and often result from first clutch failure. Focusing on first breeding attempts also allowed us to reduce the potential bias that could arise from individuals choosing a nest box that already contained a nest (as in Mingju et al., 2019). Monitoring ended when all nestlings had fledged on a given farm, which occurred between June 15 and August 5 during the study. Nest boxes were cleared of any nest material and/or dead nestlings every year in October.

### 2.3 Preference

Preference for a resource type is the likelihood of it being chosen if offered among equally available options (Johnson, 1980), and thus should ideally be assessed by choice experiments (Robertson & Hutto, 2006). However, such an approach would have been unrealistic in our case considering our large‐scale system, yearly variation, and the variety of continuous habitat characteristics we tested. We instead evaluated preference using two common surrogates that reflect the process of habitat selection: occupancy and settlement patterns (Robertson & Hutto, 2006). Preference for each nest box was estimated for each year according to the occurrence of a laying event (at least one egg laid) and settlement date (Julian date at which nesting material was first observed). Nest initiation date better represents settlement than laying date because tree swallows tend to build their nests early on and wait for clement weather to start laying (Bourret et al., 2015; Winkler et al., 2011). Laying events are thus highly synchronized and not necessarily representative of the habitat selection process. See supplemental S3 in Garrett et al. (2021a) for the phenology of breeding periods between 2006 and 2016 showing the right‐skewed laying date distribution.

**FIGURE 2:**
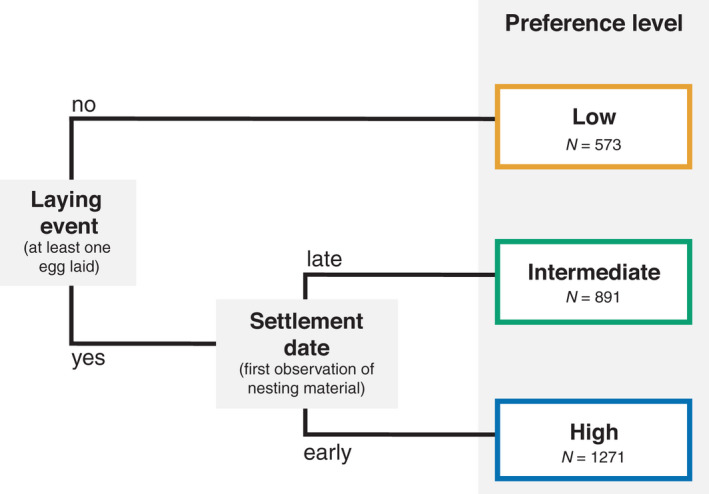
Decision tree leading to the classification of tree swallow's nest boxes in order of preference according to the presence of a laying event and the settlement date. Sample sizes (*N*) are presented for each category of preference for 400 nest boxes between 2009 and 2018, for a total of 2735 observations

Because some early settlement dates were left‐censored given that some boxes already contained nest material at the first visit (45% of all boxes), settlement dates were classified as either “early” or “late” with respect to the annual median settlement date. The category “early” included boxes with settlement dates preceding or equal to the annual median, which comprised nearly all (91.3%) left‐censored dates. Overall, the average difference between the annual mean settlement dates categorized as “early” and “late” was 10.4 ± 2.9 days (mean ± SD). Nest boxes occupied by other species were excluded from analyses (*N* = 964 boxes between 2009 and 2018). Such exclusions were made possible, even in the absence of a laying event, because the material and shape of nests are very species‐specific. We are thus confident that the vast majority of nests included in the study were initiated by tree swallows. The ordinal preference variable featured three categories ranging from least preferred (nest box hosting no laying event during the season) to most preferred nest boxes (early settlement and receiving at least 1 egg; Figure 2).

### 2.4 Habitat quality

Habitat quality was defined with respect to the fitness outcome resulting from the use of a nest site, as suggested by Johnson (2007). We used two proxies of reproductive success that, when combined, result in the number of fledglings produced during a breeding event, namely (1) the number of hatchlings produced and (2) the proportion of hatchlings that successfully fledged (i.e., fledging success).

Both the number of hatchlings and the fledging success were analyzed separately for breeding attempts of individuals that settled early and late (as defined in Figure 2), hereafter called early and late settlers, for multiple reasons. First, we know that tree swallows’ breeding success declines during the course of the season (Ghilain & Bélisle, 2008; Millet et al., 2015; Winkler & Allen, 1996) and that this decline occurs mostly between hatching and fledging in late broods (Millet et al., 2015). Because the timing of nest initiation was positively correlated with the date at which the first egg was laid (*r* = .49), late settlers should have a lower breeding success. Early and late settlers likely experience different constraints because factors that are susceptible to influence breeding performance (e.g., parasitism, food availability, or parent condition) may vary seasonally (Daoust et al., 2012; Grüebler & Naef‐Daenzer, 2010; Rioux Paquette et al., 2013). Second, in migratory birds, early arriving individuals are usually more experienced and in better conditions (Francis & Cooke, 1986; Lozano et al., 1996; Møller, 1994, 2003). Separating the effects of habitat characteristics on the breeding performance of early and late settlers hence allowed us to investigate, to some extent, the potential effects of age and body condition on the adaptiveness of nest site selection. In our system, late settlers are younger and thus less experienced (27% of females are second‐year in late settlers, versus 11% in early settlers; *G* test, *p* < .001); they are also less genetically diversified (i.e., higher internal relatedness) and have a lighter body mass than early settlers (Porlier et al., 2009). Finally, the breeding attempts of late settlers are more likely to include undetected second clutches, that is, replacement clutches laid in different nest boxes after a failure, which implies that some adults may have already invested energy in a first clutch and therefore be more limited for their second breeding attempt (Rooneem & Robertson, 1997).

Although previous studies found that the age of female tree swallows influences their breeding performance (De Steven, 1978; Rioux Paquette et al., 2014; Stutchbury & Robertson, 1988) and dispersal behavior (Lagrange et al., 2017; Winkler et al., 2004), we could not directly investigate this effect due to the imbalance of age classes (*N* = 335 clutches by second year versus *N* = 1501 by after second year) within our dataset, leading to a lack of coverage over the sampling space defined by all predictors, as well as to precision and model convergence issues.

### 2.5 Habitat characteristics

We investigated the effects of several habitat characteristics on nest box preference and reproductive success. These variables were selected based on the breeding ecology of tree swallows and included habitat characteristics describing landscape context, food availability, and both hetero‐ and conspecific social information (see details in Table 1).

#### 2.5.1 Landscape context

We characterized landscape habitat composition by measuring the relative cover of forest, of perennial forage crops, and of water bodies and wetlands, within radii of 50, 100, 200, 300, 400, 500, 1000, 5000, 10,000, and 20,000 m around each nest box. We assessed landscape habitat composition up to the 500‐m scale on a yearly basis in the field by visually identifying cultures and marginal habitats and delineating them using orthophotos (1:40,000). Characterization beyond the 500‐m scale was based on a mosaic of yearly georeferenced classified optical and radar satellite images taken between 2011 and 2018 (pixel resolution 30 m × 30 m; Agriculture and Agri‐Food Canada (AAFC), 2018). Only the year 2018 was used to assess water cover at the above range of scales because it showed better accuracy than the data of previous years (AAFC, 2020), and because the cover of large water bodies, as those covered by the data we used, did not vary significantly across years (e.g., median between‐year correlation of yearly water cover between 2011 and 2018 was 0.90 at the 10‐km scale).

Because our landscape variables were too strongly correlated to fit a multiscale model (e.g., Jedlikowski & Brambilla, 2017), we had to choose one scale for each variable. In order to use the spatial scales most representative of tree swallows’ habitat selection, we performed a preference analysis (see Statistical Analysis section) for each land cover type. Each candidate model of a model set thus included all control predictors of habitat preference used in further analyses (Table 1) in addition to the focal land cover type measured at one of the different spatial scales we considered. We then compared models on the basis of the second‐order Akaike's information criterion (AICc; Burnham & Anderson, 2002). For forest, the 100‐m spatial scale clearly had the lowest AICc score (*w* = 0.99, Figure S1a). For forage crops, all scales below 500 m were equivalent (ΔAICc < 1, Burnham & Anderson, 2002) and highly correlated (.71 ≤ *r* ≤ .97). We thus initially used a 500‐m radius to assess the effect of forage crops on habitat selection. However, tree swallows’ nest site selection also seemed to respond (yet to a lesser extent) to forage crops at the 5‐km scale (Figure S1). Because previous studies on our system found an effect of forage crops on breeding success at that scale (Ghilain & Bélisle, 2008; Porlier et al., 2009), we also run all analyses with forage crops measured at the 5‐km scale. As the 5‐km scale yielded more relevant results with respect to the questions addressed in this study, we opted to focus on this scale (see Appendix S1 for results at the 500‐m scale). We considered the interaction between forage crops at 5 km and forest cover at the same scale because forest was strongly negatively correlated with total agricultural land use, and thus open habitat (*r* = −.92, *N* = 400 nest boxes on 40 farms for 10 years). This interaction allowed us to capture the complexity of landscape contexts specific to the study system by discriminating the influence of forage crops in open versus forested landscapes. For water bodies and wetlands, two spatial scales stood out, namely the 2‐ and 10‐km radii. Since both scales yielded the same results, we used the 10‐km scale as it led to the lower AICc score (*w* = 0.20, Figure S1c).

#### 2.5.2 Food availability

Two passive insect traps were installed on each farm around the first and second third of the nest box transect. Traps consisted of ~4‐L yellow buckets placed 1.5 m above ground. They were filled with ~2 L of salty detergent solution to reduce surface tension and slow the growth of bacteria and fungi. Two transparent plexiglass screens were mounted perpendicularly to one another above each bucket to intercept flying insects (see Bellavance et al., 2018 and Garrett et al., 2021a for details). We collected the content of each trap on every visit to a farm (i.e., every other day) and conserved arthropods in 70% ethanol until processing. We sorted samples by removing arthropods unlikely to be preyed upon by tree swallows (i.e., bumblebees (*Bombus* spp.: Hymenoptera), June bugs (*Phyllophaga* spp.: Coleoptera), large spiders (Araneae, >0.5 cm body width), and caterpillars (Lepidoptera); Bellavance et al., 2018). The rest of the sample was dried at 50°C for at least 48 h before being weighed (Adam Equipment, model AAA250L, ±0.0001 g). The mean daily dry biomass of arthropods collected between May 1 and May 15 was used as a proxy of yearly food availability on a given farm at the time of nest site selection in further analyses. We compared the average daily insect biomass collected during this period with that collected during the nestling period, defined by the farm's yearly average hatching date and the following 12 days (yearly mean hatching date on the system was used for farms with no nestlings). The correlation between the insect biomass of the two periods was then calculated for each farm in order to assess within‐season predictability of this food resource.

#### 2.5.3 Heterospecific social information

House sparrows (*Passer domesticus*) are tree swallow's main nest site competitors in our system, and they initiate breeding before swallows return from their wintering grounds (Robillard et al., 2013). We evaluated the use of heterospecific social information through the number of nest boxes occupied by house sparrows on each farm in the current year. Nest boxes and house sparrow nests were visited every other day concurrently to tree swallow monitoring. Occupancy was determined by the presence of at least one egg, and only first clutches observed in each box were included since a nest box is rarely used by another species once house sparrows have built a nest therein.

#### 2.5.4 Conspecific social information

We defined two sources of social information regarding the future breeding success that an individual could expect to experience on a given farm: the density of tree swallows that bred on a farm during the previous year and the mean number of fledglings obtained by those breeders (Lagrange et al., 2017). This information can be collected directly by an individual breeding on the farm during the previous year, or by prospecting individuals that explored habitats either during or after the previous breeding season. We worked at the farm level because Lagrange et al. (2014) found that fidelity was high at this scale within our study system: The probability of a female breeding on the same farm for two consecutive years varied between 70% and 94% depending on the occurrence of a dispersal event the year before. However, we found that the probability of a female reproducing in the same nest box over two consecutive years between 2009 and 2018 was only 5%. This suggests that a significant component of habitat selection occurs at the farm rather than at the nest box level. This is coherent with the fact that tree swallows are semi‐colonial and highly mobile, traveling in our study system regularly up to 15 km (Lessard et al., 2014) and 10 km (Garrett et al., 2021b) to find a mate and to forage when provisioning food to their nestlings, respectively.

### 2.6 Statistical analyses

To determine whether settlement decisions deviate from an ideal habitat selection, we compared the relationships linking habitat characteristics to habitat preference and reproductive success. Specifically, if the relationship between habitat preference and a given habitat characteristic was qualitatively similar to the one linking breeding success to that characteristic, we concluded to a case of ideal settlement decision. We identified nonideal decisions when there was a mismatch or uncoupling between those two relationships. For example, a habitat characteristic that was preferred but that was unrelated to reproductive success suggested a case of nonideal selection. Alternatively, a characteristic that was not preferred but that influenced reproductive success also suggested a nonideal habitat selection (“equal‐preference trap”; Robertson & Hutto, 2006). Lastly, if both relationships went in opposite directions, habitat selection was considered even more maladaptive, and potentially indicating an ecological trap (Pärt et al., 2007).

#### 2.6.1 Habitat predictability

All analyses were performed in the R environment (v. 3.5.3, R Core Team, 2019). For cues to provide information about the expected breeding success of a given nesting site, they must show some correlation in time (Doligez et al., 2003). We thus assessed the between‐year predictability of habitat characteristics by determining the correlation between the current‐ and previous‐year values of habitat characteristics of the 400 nest boxes. For the year 2010 to 2018, we computed Pearson's correlation coefficient. We then used the mean of those annual correlation coefficients and the standard deviation to assess between‐year predictability of every habitat characteristic studied, except water bodies and wetlands for which we only used the data from 2018.

#### 2.6.2 Preference

Ordinal logistic regression was used to model the preference for nest boxes with the *ordinal* package (v. 4‐25, Christensen, 2019) using a three‐category ordinal response variable based on nest box occupancy and settlement date (Figure 2). The proportional odds assumption, which states that the coefficient of each predictor should be constant between all pairs of response categories, was found to hold according to the graphical approach suggested by Harrell (2015). We built a series of models that included all combinations of the groups of variables that characterized landscape context, food availability, and both hetero‐ and conspecific social information, as described and justified in Table 1. All models also included variables controlling for geographical position (latitude and longitude) and spring weather. See Table A1 for the list of candidate models. We found no evidence of problematic multicollinearity among predictors as variance inflation factors were all below 3 (Zuur et al., 2009). See Figure S2 for the matrix of correlations of all predictors. Predictors were standardized (zero mean, unit variance). Random effects included nest box, farm, and year identity. We compared models based on AICc with the *AICcmodavg* package (v 2.2‐2, Mazerolle, 2019). Because the weight of evidence of the best model for this analysis was strong (*w* = 0.88), we decided to base our inferences of nest box preference on that single model (see Table A2 for results of model selection).

#### 2.6.3 Habitat quality

All analyses of reproductive success were performed with the *glmmTMB* package (v. 0.2.3; Brooks et al., 2019). We compared the same list of candidate models used for the preference analysis (Table A1) based on AICc in order to assess whether the determinants of nest box preference are linked to reproductive success. Because no one model clearly stood out above others, we performed multimodel inference for generating predictions (and unconditional 95% confidence intervals) following Burnham and Anderson (2002). We tested for zero‐inflation of both the number of hatchlings and fledging success using the *DHARMa* package (Hartig, 2019).

Number of hatchlings was modeled with zero‐inflated generalized linear mixed models (GLMM) using a generalized Poisson distribution with a log link function for the conditional model and a logit link function for the zero‐inflated model (Brooks et al., 2019). Both the conditional and zero‐inflated models contained the same fixed effects, but not the same random effects. Indeed, we added the identity of the combination of year and farm as a random effect in the zero‐inflated model to account for sporadic events that can occur on a farm and lead to the failure of nearly all clutches, and that we may have not detected or measured (e.g., disturbance by a predator causing birds to abandon their clutch). Also, because of convergence issues, we could not keep all random effects and removed those that accounted for very little variance (i.e., <1 × 10^−7^% of the variance explained by random effects). We thus removed the year in the early settlers’ conditional model and both nest box and farm identity in the zero‐inflation model. For late settlers, we had to remove farm identity in the conditional and zero‐inflation models along with year identity in the zero‐inflation model to reach full convergence. Those changes did not affect the magnitude nor the precision of the parameter estimates. We modeled fledging success as a proportion of hatchlings having successfully fledged with GLMM using a binomial error distribution and logit link function. Random effects included nest box, farm, and year identity.

## 3 RESULTS

### 3.1 Habitat predictability

Habitat characteristics were generally predictable between years. Predictability was especially high for forest cover within 100 m and 5 km (0.96 ± 0.04 and 0.99 ± 0.01, mean annual correlation coefficient between current and previous year value ±SD), forage crop cover within 5 km (0.91 ± 0.10), house sparrow density (0.85 ± 0.04), and tree swallow density (0.85 ± 0.03). Predictability was moderate for spring insect biomass (0.41 ± 0.22) and mean number of fledglings on a farm (0.25 ± 0.15). Finally, we found that the predictability of insect biomass between the time of habitat selection and nestling food provisioning was low (*r* = .29 ± .34, mean of all 40 farms ±SD) and highly spatially variable (−.45 ≤ *r* ≤ .89, depending on farms).

### 3.2 Preference

On average, 64.3% ±7.5 (mean ± SD) of our 400 nest boxes were annually occupied by tree swallows, 16.6% ±6.4 by house sparrows, and 2.3% ±1.5 by other bird species (i.e., house wrens (*Troglodytes aedon*), eastern bluebirds (*Sialia sialis*), and black‐capped chickadees (*Poecile atricapillus*)). Each year on average, 12.7% ±3.1 nest boxes received nesting material but no laying event, while 6.8 ± 2.5 stayed empty of nesting material throughout the season.

**FIGURE 3:**
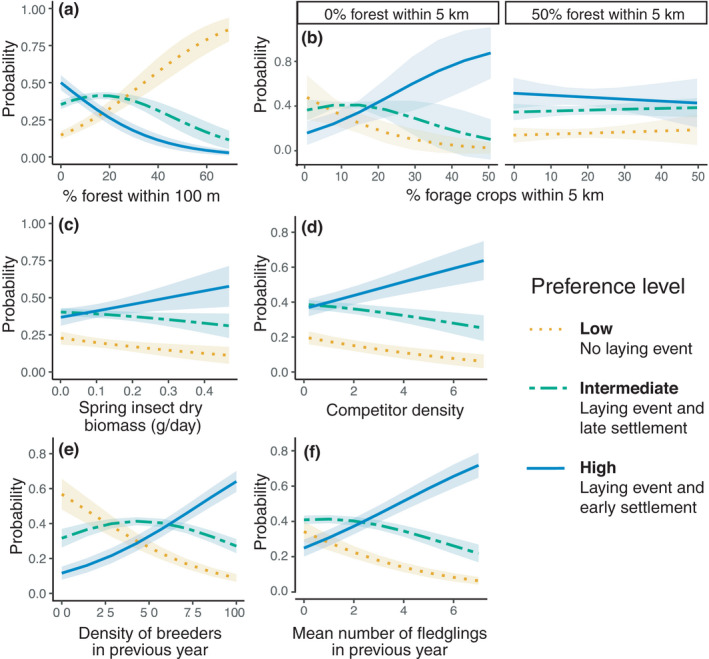
Predicted probabilities of a tree swallow nest box being classified in low, intermediate, or high preference level (as defined in Figure 2) in southern Québec, Canada, between 2009 and 2018, as a function of (a) forest cover near nest box, (b) agricultural intensity and openness of landscape, (c) food availability in spring, (d) competitor density, (e) density of breeders on a farm in the previous year, and (f) mean performance of breeders (number of fledglings) in the previous year. Inference was based on an ordinal mixed logistic regression (model #16 in Table A1). See Table A2 for details on model selection. Other variables in the model were kept at their average value. Shaded areas represent 95% confidence intervals. *N* = 2915 potential breeding attempts along 10 years on 40 farms

We found a relationship to preference (Figure 2) for almost all habitat characteristics describing either the landscape context, food availability, or both hetero‐ and conspecific social information (Table 2). Regarding landscape composition, preference decreased with forest cover within a 100‐m radius, and thus, the probability of tree swallows laying in highly forested habitats was very low (Figure 3a). The effect of the cover of forage crops within 5 km depended on forest cover (Figure 3b). Individuals preferred nest boxes surrounded by high proportions of forage crops in sparsely forested landscapes, but forage crops had little influence on preference when forest cover reached ≥50% of the measured area. The amount of wetlands and open water within 10 km was the only landscape composition variable not related to nest box preference (Table 2). As expected, nest box preference increased with spring food availability (Figure 3c). Contrary to our expectations, preference was positively correlated with house sparrow density (Figure 3d). As for conspecific social information, preference increased with both the density of breeding tree swallows on a farm and their reproductive performance in the previous year (Figure 3e–f).

**TABLE 2:** Determinants of nest box preference in tree swallows and their effect on the reproductive success of early and late settlers in a nest box network in southern Québec, Canada, between 2009 and 2018

Explanatory variable	Estimate (95% confidence interval)						
	Preference *N* = 2915	Number of hatchlings				Fledging success	
		Early settlers *N* = 1268		Late settlers *N* = 891		Early settlers *N* = 953	Late settlers *N* = 644
		Conditional	Zero‐inflated	Conditional	Zero‐inflated		
Forest 100 m	**−0.58 (−0.70, −0.46)**			−0.01 (−0.03, 0.01)	0.12 (−0.04, 0.29)	0.10 (−0.07, 0.28)	**−0.49 (−0.71, −0.27)**
Forest 5 km	0.21 (−0.07, 0.48)			**0.05 (0.01, 0.08)**	−0.07 (−0.39, 0.25)	−0.44 (−0.93, 0.06)	0.06 (−0.35, 0.48)
Forage crops 5 km	0.15 (−0.04, 0.34)			0.00 (−0.04, 0.03)	−0.21 (−0.51, 0.09)	**0.53 (0.26, 0.80)**	−0.03 (−0.33, 0.27)
Forest 5 km × Forage crops 5 km	**−0.28 (−0.50, −0.06)**			−0.03 (−0.06, 0.01)	**0.61 (0.27, 0.95)**	−0.58 (−0.90, −0.26)	−0.16 (−0.48, 0.16)
Water 10 km	−0.11 (−0.37, 0.15)			−0.01 (−0.04, 0.02)	−0.16 (−0.47, 0.15)	**0.57 (0.13, 1.01)**	−0.20 (−0.60, 0.20)
Insect biomass	**0.12 (0.01, 0.24)**						
Competitor density	**0.26 (0.13, 0.38)**	−0.01 (−0.03, 0.01)	**0.22 (0.02, 0.41)**	−0.01 (−0.03, 0.01)	**0.24 (0.06, 0.42)**		
Density @ t−1	**0.69 (0.55, 0.82)**	**0.02 (0.00, 0.04)**	−0.15 (−0.35, 0.05)			−0.02 (−0.19, 0.15)	**−0.45 (−0.64, −0.26)**
Success @ t−1	**0.39 (0.30, 0.48)**	−0.01 (−0.02, 0.01)	−0.16 (−0.33, 0.02)			**−0.25 (−0.38, −0.13)**	**0.40 (0.25, 0.55)**
Temperature	−0.01 (−0.15, 0.14)	−0.01 (−0.03, 0.00)	0.24 (−0.08, 0.56)	−0.01 (−0.05, 0.02)	−0.04 (−0.23, 0.16)	0.25 (−0.04, 0.55)	−0.16 (−0.63, 0.31)
Precipitations	**−0.15 (−0.24, −0.07)**	0.01 (0.00, 0.03)	0.01 (−0.17, 0.19)	0.01 (−0.01, 0.03)	−0.16 (−0.34, 0.02)	0.05 (−0.06, 0.16)	−0.02 (−0.15, 0.10)
Longitude	−0.24 (−0.53, 0.04)	0.01 (−0.01, 0.03)	0.10 (−0.09, 0.28)	−0.01 (−0.05, 0.03)	0.26 (−0.09, 0.61)	−0.19 (−0.68, 0.30)	0.07 (−0.36, 0.50)
Latitude	0.00 (−0.22, 0.21)	**−0.02 (−0.04, 0.00)**	0.04 (−0.13, 0.21)	0.03 (0.00, 0.05)	−0.21 (−0.46, 0.04)	0.22 (−0.15, 0.59)	**−0.39 (−0.73, −0.05)**

Note

Coefficients come from an ordinal logistic mixed regression for preference, a zero‐inflated GLMM using generalized Poisson distribution and log link function for the number of hatchlings and GLMM using binomial error distribution and logit link function for fledging success. Predictors were standardized (zero mean, unit variance). For each analysis, we present the coefficients of the model that ranked best in terms of AICc, their 95% confidence intervals, and the sample size. See Table 1 for definitions of the variables and Table A2 for results of the model selection. Estimates for which the confidence interval excludes zero are in bold.

### 3.3 Habitat quality

#### 3.3.1 Number of hatchlings

Overall, 33% of early settlers’ clutches experienced complete hatching failure, compared with 38% for late settlers. Hatching failure was either caused by predation or from an unexplained interruption of incubation. Unexplained interruption of incubation was the most common cause of hatching failures in both early and late settlers (66% and 58% of failures, respectively). Mean number of hatchlings (±SD) for early and late settlers that did not experience hatching failure was 5.0 ± 1.2 and 4.7 ± 1.3, respectively.

For early settlers, the model including both hetero‐ and conspecific social information best described the number of hatchlings (*w* = 0.33; Table A2). Number of hatchlings increased with tree swallow density on the farm in the previous year, while the probability of hatching failure increased with house sparrow density (Table 2). Those effects were also found in the second best model (*w* = 0.22; Table A3), while the third best model (*w* = 0.16; Table A3), which did not include conspecific social information, also showed a negative effect of house sparrow density on the number of hatchlings.

**FIGURE 4:**
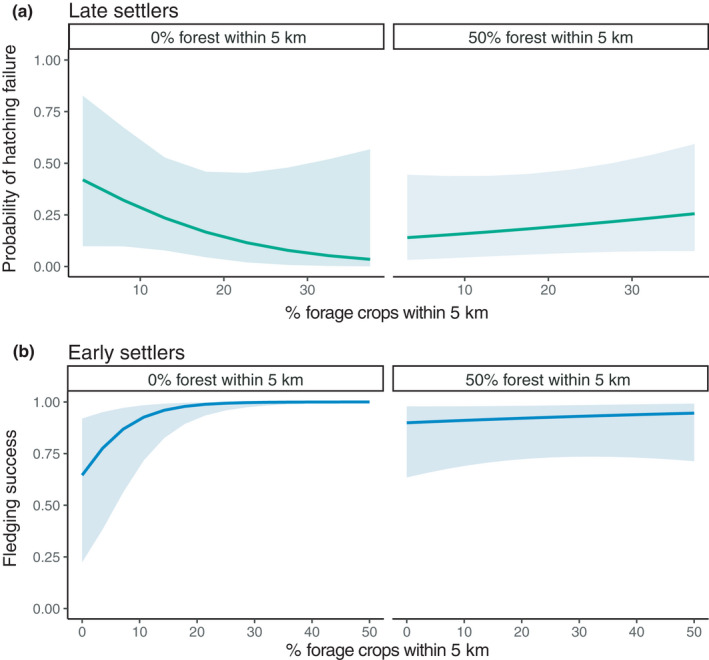
Averaged predicted (a) probability of hatching failure of late settlers and (b) fledging success of early settlers as a function of agricultural intensity and openness of landscape, for tree swallows in a nest box network in southern Québec, Canada, between 2009 and 2018. Multimodel inference was made on the list of models presented in Table A1; see Table A2 for the Akaike weights. Other variables in the model were kept at their average value. Blue = early settlers, *N* = 953. Green = late settlers, *N* = 644

Late settlers’ number of hatchlings was best described by the model including landscape context and heterospecific social information (*w* = 0.27; Table A2). The number of hatchlings increased with forest cover at 5 km, and the probability of hatching failure decreased with forage crop cover within 5 km where forest cover was low (Table 2; Figure 4a). Probability of hatching failure increased with high house sparrow density (Table 2). The third best model also included these effects (*w* = 0.14, Table A3). However, the second best model only included conspecific social information variables (*w* = 0.11, Table A3), and showed higher number of hatchlings where the density of swallows in the previous year was high, along with a lower probability of hatching failure where the mean number of fledglings in the previous year was high (as also seen when using the 500‐m scale for forage crops; Table A6).

#### 3.3.2 Fledging success

The average proportion of nestlings that fledged was 0.75 ± 0.37 and 0.69 ± 0.41 (mean ± SD) for early and late settlers, respectively. The model including both landscape context and conspecific social information best described the fledging success of both early and late settlers (*w* = 0.42 and 0.26, Table A2).

For early settlers, fledging success was higher where the cover of water within 10 km was high and where the cover of forage crops within 5 km was high, this effect being stronger in open landscapes (Figure 4b). Fledging success also decreased slightly with the mean number of fledglings produced on the farm in the previous year (Figure S3). All of these effects were also found in the second and third best models (*w* = 0.27 and 0.20; Table A4).

For late settlers, fledging success decreased with forest cover within 100 m and with the prior year density of tree swallows. On the contrary, it increased with the mean number of fledglings produced on the farm in the previous year (Figure S3). Those effects were also found in the second and third best models (*w* = 0.25 and 0.25; Table A4).

## 4 DISCUSSION

We investigated the links between several environmental and social habitat characteristics, nest box preference, and annual reproductive success in a tree swallow population breeding along a gradient of agricultural intensification. We found multiple mechanisms that may lead to an ecological trap, which took two forms: (1) a habitat characteristic that was associated with preference, but not with reproductive success (or the other way around); and, more severely, (2) a habitat characteristic for which the relationship between preference and reproductive success went in opposite directions. Some environmental cues, such as spring food availability and water bodies, led to nonideal habitat choices of the first form. Contrary to our hypothesis, we found occurrences where both hetero‐ and conspecific social information promoted mismatches of the second form. Moreover, some landscape features led to ideal habitat selection patterns. Our study thus did not show that environmental cues were poorer predictors of habitat quality than social cues, but it did highlight that some settlement decisions drive our tree swallow population further away from an ideal distribution. Our results have implications for declining farmland birds and for the use of nest boxes as a conservation tool.

### 4.1 Landscape

Forest cover within 100 m was associated with ideal habitat selection decision making since nest boxes surrounded by more forest were less attractive to tree swallows and also led to a lower fledging success in late settling individuals. Previous studies found that tree swallows avoid breeding near forest, both in a nest box grid and in natural tree cavities (Rendell & Robertson, 1990; Robles & Martin, 2013). Breeding near forest edges may be avoided because it (1) requires individuals to travel farther to forage (Bruun & Smith, 2003), and/or (2) impedes nest defense against predators (Rendell & Robertson, 1990), which may be more active and/or abundant along forest edges (Chalfoun et al., 2002; Lahti, 2001). These limitations may be more important for late settlers because they are on average less experienced and in worse condition than early settlers, and thus potentially less able to cope with increased foraging costs (Frey‐Roos et al., 1995). Flying insects may also be less abundant as the season progresses, making foraging more costly for late settlers (Bellavance et al., 2018; Garrett et al., 2021a; Rioux Paquette et al., 2013).

Forage crops were preferred by tree swallows only in landscapes mostly denuded of forest cover. In our system, forage crops are generally cultivated over much smaller areas and within more forested areas than intensive row crops (Bélanger & Grenier, 2002). It may then be that forage crops are preferred in landscapes where open habitat is less fragmented by forest. Forage crops are also likely preferred because, contrary to annual row crops, they green up earlier and may harbor higher insect densities in early spring (Thorup et al., 2017). This being said, wind‐protected vegetated boundaries often support higher insect densities (Grüebler et al., 2008; McCarty & Winkler, 1999, 1999) and can provide prime foraging opportunities to aerial insectivores (Evans et al., 2003; Stanton et al., 2016). We also found that, in open landscapes, forage crops increased early settlers’ fledging success and decreased late settlers’ probability of hatching failure, suggesting adaptive habitat selection (Figure 4). These results are coherent with previous studies that found a positive impact of forage crops on breeding performance in our study area (Daoust et al., 2012; Ghilain & Bélisle, 2008; Porlier et al., 2009).

We did not find that water and wetland cover within 10 km was a determinant of nesting site preference, which was surprising considering that tree swallows typically breed near water (Winkler et al., 2011) and feed on insects with an aquatic larval stage, even in our study area (Bellavance et al., 2018; Elgin et al., 2020; McCarty & Winkler, 1999, 1999; Michelson et al., 2018). However, we did find that early settlers’ fledging success increased in landscapes where water bodies and wetlands were more abundant (Table 2). This lack of preference for an important predictor of habitat quality suggests nonideal habitat selection. However, it is important to note that our ability to detect potential effects of water was limited by the low availability of these habitats in our study area (range: 1–11% within 10 km). Yet, our result does stress the well‐known importance of water bodies and wetlands for breeding tree swallows (Berzins et al., 2021).

### 4.2 Food availability

Several studies found that prey availability affects breeding habitat selection in insectivorous birds (Burke & Nol, 1998; Petit & Petit, 1996), including aerial insectivores (Brown & Brown, 1996; English et al., 2017; Forsman et al., 1998). Tree swallows were found to settle preferentially in habitats showing higher spring insect availabilities and this, despite that insect biomass during nest site selection was poorly correlated to that of the food provisioning period. The fact that spring insect availability was a poor indicator of future foraging conditions within an agricultural context is not surprising given that pesticide applications and other farming activities can unpredictably disrupt insect phenology and abundance (Botías et al., 2019; Mulé et al., 2017; Pisa et al., 2015). This observation concords with previous studies that found important between‐year and within‐season differences in Diptera and total insect abundance patterns along the agricultural intensification gradient of our study area (Bellavance et al., 2018; Garrett et al., 2021a; Rioux Paquette et al., 2013). Given its low within‐season predictability, it is also not surprising that we observed no relationship between spring insect availability and the breeding performance metrics we considered. While our result does not by any means downplay the importance of food availability for feeding nestlings (Garrett et al., 2021a, 2021b; McCarty & Winkler, 1999, 1999; Nooker et al., 2005), it nevertheless leads us to conclude that food availability at the time of nest site selection is a nonideal settlement cue for these birds when breeding in current agroecosystems.

### 4.3 Heterospecific social information

Tree swallows were attracted to farms presenting high house sparrow densities, their main competitors for nest sites. We are confident that this trend is not an artifact of nests being initiated by house sparrows since the effect was still present if we measured preference based on tree swallows laying date instead of settlement (nest initiation) date. Swallows that settled on such farms were more likely to experience hatching failure, an effect seen in both early and late settlers (Table 2). Such an increased hatching failure probably results from competition for nest boxes between these two species often leading to lethal interactions, nest usurpation, and destruction of swallows’ eggs (Winkler et al., 2011). Given the significant fitness costs imposed by such agonistic interactions, we may expect tree swallows to use the abundance of house sparrows for detecting breeding habitat and assessing its quality, as information provided by heterospecifics is often used for such purposes (Forsman et al., 2002; Kivelä et al., 2014; Mönkkönen et al., 1990; Parejo et al., 2008; Thomson et al., 2003). For time‐limited species such as migrants, the presence of a resident species sharing some ecological requirements or mortality factors, such as house sparrows for tree swallows, could be a useful indicator of habitat quality (Mönkkönen et al., 1999; Parejo et al., 2005; Seppänen et al., 2007; Thomson et al., 2003). Unlike conspecific social information, it is often available upon arrival on the breeding grounds and also available for individuals who had no access to previous‐year information (i.e., dispersers; Doligez, 2002; Kivelä et al., 2014). Moreover, heterospecific attraction is more likely to occur when search costs are high, which is likely the case for cavity users due to the scarcity of nest sites, but only when the costs of competition are low (Mönkkönen et al., 1999; Seppänen et al., 2007; Stamps et al., 2005).

Given the above, it is thus surprising that tree swallows were attracted to sites where they incurred greater costs from house sparrows. One potential explanation for this result is that tree swallows’ habitat preferences likely evolved with less aggressive nest site competitors (e.g., black‐capped chickadees). House sparrows were introduced from Europe to the United States in the 1850s (Lowther & Cink, 2006), and competition with tree swallows was likely exacerbated by agricultural intensification over the last decades through their access to farm buildings for nesting sites and grains as a food resource (Robillard et al., 2013). Although competitors for cavities are generally more abundant in natural environments partly due to the larger entrance of natural cavities as compared to nest boxes (Norris et al., 2018; Robertson & Rendell, 1990), they may also be less aggressive than house sparrows (Winkler et al., 2011).

### 4.4 Conspecific social information

We found that tree swallows used conspecific social information as settlement cues, but the relationship to both the density of conspecifics and their breeding success in the previous year yielded contradictory results. For early settlers, density of breeders in the previous year seemed adaptive while fledging success in the previous year seemed maladaptive on the basis of number of hatchlings and fledging success, respectively. The opposite conclusions were observed for late settlers based solely on fledging success. Thus, both early and late settlers were susceptible to being trapped into breeding on farms where they would experience lower fledging success, yet based on different sources of social information.

Many studies have shown that individuals could be attracted to habitats occupied by conspecifics (e.g., Nocera et al., 2006; Stamps, 1988; Ward & Schlossberg, 2004). Conspecific attraction, by causing individuals to breed in aggregation, has many potential benefits, including: increased detectability of both nesting and foraging habitat patches (Barta & Giraldeau, 2001; Brown, 1988; King & Cowlishaw, 2007; Stamps, 2001), increased mating and extra‐pair copulation opportunities (Griffith et al., 2002; Lessard et al., 2014), and increased detectability and defense against predators (Smith, 1986; Turner & Pitcher, 1986). Breeding in aggregation can also bring density‐dependent costs such as aggressive interactions and competition for resources (Fretwell & Lucas, 1970; Newton, 1998; Sutherland, 1996; Winkler et al., 2011), and increased predation and parasitism (Møller, 1989). Here, we found that these costs might overcome the benefits of cueing on conspecifics density for late settlers, who experienced a lower fledging success on (previously) densely populated farms.

Two previous studies in our system showed the importance of public information on settlement decisions by finding that a given nest box occupancy was positively correlated to the fledging success experienced in the same box in the previous year (see Ghilain & Bélisle, 2008; Robillard et al., 2013). In this study, we showed that this trend also occurred at the farm level, even though the predictability of breeding success was moderate. Public information regarding reproductive success is thought to be the most robust and integrative form of social information about the consequences of local environmental factors on this fitness component (Doligez et al., 2003; Seppänen et al., 2007; Valone & Templeton, 2002). Yet, we found that public information led to a slight but significant mismatch between habitat preference and quality in early settlers, whose fledging success decreased with the mean number of nestlings fledged on the farm in the previous year. Nevertheless, early settlers’ fledging success remained higher than that of late settlers (Figure S3) and this potential ecological trap may only have a limited impact on population dynamics. The mechanism underlying this putative trap is unclear but is likely to result from one or more factors that were not measured in this study and that are correlated to past breeding success rather than to breeding success itself, or from a statistical artifact whereby high annual breeding success estimates punctually “regress to the mean” (Barnett, 2004).

### 4.5 Temporal constraints

Relationships between cues and breeding success varied according to the timing at which tree swallows settled in nest boxes, which underlines the relevance of considering different behavioral adaptive outcomes for individuals that may experience different constraints when studying habitat selection. Indeed, we showed that early and late settlers faced different selective pressures along the breeding season likely due to differential experience, body condition or timing (breeding phenology). At our latitudes, time constraints can induce such interindividual disparity because breeding conditions peak in quality over a short period and may thereby lead to phenological mismatches (Bourret et al., 2015; Visser & Gienapp, 2019). Moreover, individuals that settle later benefit from less nest site options and, assuming they arrive later on breeding grounds, may also have less time for exploration, which can lead to decisions based on poor or incomplete knowledge (Orians & Wittenberger, 1991). Such time‐limited exploration has indeed been hypothesized to cause migratory red‐backed shrikes (*Lanius collurio*), which prey upon insect in open habitat to fall into an ecological trap in agroforested landscapes of NW Europe (Hollander et al., 2013). While late settlers often make poorer habitat choices due to time constraints, we also detected mismatches between preference and habitat quality in early settlers due to competition. Our results emphasize the ecological importance of phenological (Visser & Gienapp, 2019) and phenotypic (Edelaar et al., 2008; Matthysen, 2012) (mis)matches and support the hypothesis that there may be costs to breed either too early or too late. Such costs may become particularly important for ground‐nesting farmland birds as climate change can affect differently their breeding phenology and the timing of sowing and harvesting and thus the presence of ground cover for nests and their risk of being destructed by farm tools (Santangeli et al., 2018).

It is worth mentioning that our estimate of nest site preference is imperfect. Settlement patterns may not directly represent habitat preference if there are alternative selection strategies among individuals (e.g., within and among age classes Robertson & Hutto, 2006). Yet, our three‐category estimate of preference, integrating two rather than typically just one surrogate of habitat preference, should allow a good understanding of the habitat selection process used by tree swallows as it discriminates between the choice of a nest site and the timing of that choice. Interestingly, it is not excluded that the preference patterns we observed have a genetic component, especially since tree swallows from our study system show a spatial genetic structure for a candidate gene related to the timing of migration in passerines. The settlement patterns we observed may thus have a genetic basis (Bourret & Garant, 2015).

Our proxies of habitat quality, that is the number of hatchlings and fledging success, only represent the reproductive success at the nesting stage. Because we did not investigate adult survival, post‐fledging survival, or recruitment rate, our habitat quality proxies are not perfectly representative of fitness experienced by individuals using a given habitat (Johnson, 2007), especially since post‐fledging and adult survival likely depend on physiological, phenological, and environmental factors (Boynton et al., 2020; Clark et al., 2018; Evans et al., 2020; Greño et al., 2007; Naef‐Daenzer et al., 2001). Nonetheless, fledging success and number of fledglings are among the most important determinants of population growth and lifetime reproductive success in this species (Berzins et al., 2020; Cox et al., 2018). More research is needed to determine the demographic consequences of the nonideal behaviors we identified and their potential impact on long‐term population growth. For instance, assessing demographic rates associated with different habitat types in order to investigate whether local tree swallow populations of our study area are subjected to a source–sink dynamic resulting from an ecological trap would be a valuable complement to the current study. Moreover, it would be interesting to compare the habitat selection of populations breeding in nest boxes with those using natural cavities since these two types of nest sites are usually associated with very different ecosystems, natural cavities being mostly found in areas that are less subjected to human perturbation.

We found that landscape context, spring food availability, and social information from both hetero‐ and conspecifics influence tree swallows’ nest site preference. Relying on multiple cues for assessing habitat quality may render this species less susceptible to making bad habitat choices and fall into a severe ecological trap (Hale et al., 2015; Pärt et al., 2011; Valone & Templeton, 2002). Yet, by investigating the relationships between habitat characteristics and quality, we also identified several settlement decisions suggestive of ecological traps. Contrary to our hypothesis, we found the most severe mismatches between nest site preference and fitness outcome to be associated with the use of hetero‐ and conspecific social information. Given these results, there is thus evidence that farmlands can potentially lead to an ecological trap via different mechanisms, including low within‐season insect predictability, the presence of house sparrows as nest site competitors, and nest boxes creating supra‐optimal densities. This has some implications regarding the decline of farmland birds, which has been attributed to habitat loss and alteration, interference with farming equipment, and direct (toxicological) and indirect (trophic) effects of pesticide use (Stanton et al., 2018). Moreover, our study has implications for the use of nest boxes as a conservation and research tool (Lambrechts et al., 2010; Møller & Moller, 1992). Indeed, natural cavities are often limited in numbers, and artificial ones can be provided with the potential consequence of attracting birds into novel or improper breeding habitats (Holt & Martin, 1997; Maícas et al., 2012; Newton, 1998).

## CONFLICT OF INTEREST

None declared.

## AUTHOR CONTRIBUTION

**Ève Courtois:** Conceptualization (equal); Formal analysis (lead); Investigation (lead); Methodology (lead); Validation (lead); Visualization (lead); Writing‐original draft (lead); Writing‐review & editing (equal). **Dany Garant:** Funding acquisition (equal); Project administration (equal); Resources (equal); Supervision (supporting); Writing‐review & editing (supporting). **Fanie Pelletier:** Funding acquisition (equal); Project administration (equal); Resources (equal); Writing‐review & editing (supporting). **Marc Bélisle:** Conceptualization (equal); Funding acquisition (equal); Methodology (supporting); Project administration (equal); Resources (equal); Supervision (lead); Writing‐review & editing (equal).

## Supporting information

Fig S1

Fig S2

Fig S3

Fig S4

Appendix S1

Supplementary Material

## Data Availability

Full dataset is archived in Dryad: Courtois, Ève et al. (2021), Nonideal nest box selection by tree swallows breeding in farmlands: evidence for an ecological trap?, Dryad, Dataset, https://doi.org/10.5061/dryad.2bvq83brd
