# Supplementary material for: Nonideal nest box selection by tree swallows breeding in farmlands: Evidence for an ecological trap?
Source: Ecol Evol. 2021 Nov 9;11(22):16296–313. doi: 10.1002/ece3.8323 (PMC8601888; doi:10.1002/ece3.8323)
Supplement: Supplementary file 5 — Appendix S1 [file ECE3-11-16296-s002.docx]

Appendix

Complementary data

Some of our explanatory variables, notably landscape-related ones, showed correlations (Fig. A2). High forest cover within 5 km was associated with high cover of forage crops within 5 km (*r* = 0.66) and high cover of water bodies and wetlands within 10 km (*r* = 0.68), which in turn was associated with high cover of forage crops in 5 km (*r* = 0.53). Those landscape variables were also associated with a longitudinal gradient and, to a lesser extent, latitudinal gradient. Forest within 5 km, forage crops within 5 km and water within 10 km were more abundant in the eastern part of the system (*r* = 0.71, 0.71 and 0.63) and in the South (*r* = -0.38, -041 and -0.51). Finally, a high density of house sparrow on a farm was associated with a low density of tree swallow in the previous year (*r* = -0.50).

Table A1. List of the candidate models used to assess the determinants of habitat preference and their impact on the reproductive success of tree swallows in a nest box network in southern Québec, Canada, between 2009 and 2018.

| # | Candidate model |
| --- | --- |
| 0 | ~ Null |
| 1 | ~ Control |
| 2 | ~ Landscape + Control |
| 3 | ~ Food + Control |
| 4 | ~ Heterospecific social information + Control |
| 5 | ~ Conspecific social information + Control |
| 6 | ~ Landscape + Food + Control |
| 7 | ~ Landscape + Heterospecific social information + Control |
| 8 | ~ Landscape + Conspecific social information + Control |
| 9 | ~ Food + Heterospecific social information + Control |
| 10 | ~ Food + Conspecific social information + Control |
| 11 | ~ Heterospecific social information + Conspecific social information + Control |
| 12 | ~ Landscape + Food + Heterospecific social information + Control |
| 13 | ~ Landscape + Food + Conspecific social information + Control |
| 14 | ~ Landscape + Heterospecific social information + Conspecific social information + Control |
| 15 | ~ Food + Heterospecific social information + Conspecific social information + Control |
| 16 | ~ Landscape + Food + Heterospecific social information + Conspecific social information + Control |

Landscape, food, heterospecific and conspecific social information, and control refer to the categories of predictors presented in Table 1.

| Table A2. Results of the model selections made on the candidate models presented in Table A1 for the different response variables: nest box preference, number of hatchlings and fledging success of tree swallows in southern Québec, Canada, between 2009 and 2018. | | | | | | | | | | | | | | | |
| --- | --- | --- | --- | --- | --- | --- | --- | --- | --- | --- | --- | --- | --- | --- | --- |
| Candidate model | Preference | | | Number of hatchlings | | | | | | Fledging success | | | | | |
|  |  |  |  | Early settlers | | | Late settlers | | | Early settlers | | | Late settlers | | |
|  | K | ΔAICc | *w* | K | ΔAICc | *w* | K | ΔAICc | *w* | K | ΔAICc | *w* | K | ΔAICc | *w* |
| 0 | 5 | 289.72 | 0.00 | 7 | 24.39 | 0.00 | 7 | 2.49 | 0.08 | 4 | 23.02 | 0.00 | 4 | 54.63 | 0.00 |
| 1 | 9 | 281.68 | 0.00 | 15 | 20.30 | 0.00 | 15 | 9.30 | 0.00 | 8 | 26.57 | 0.00 | 8 | 57.28 | 0.00 |
| 2 | 14 | 151.51 | 0.00 | 25 | 27.96 | 0.00 | 25 | 3.53 | 0.05 | 13 | 11.70 | 0.00 | 13 | 45.91 | 0.00 |
| 3 | 10 | 283.09 | 0.00 | 17 | 20.37 | 0.00 | 17 | 12.05 | 0.00 | 9 | 26.96 | 0.00 | 9 | 54.34 | 0.00 |
| 4 | 10 | 283.18 | 0.00 | 17 | 1.42 | 0.16 | 17 | 7.50 | 0.01 | 9 | 28.59 | 0.00 | 9 | 59.33 | 0.00 |
| 5 | 11 | 121.81 | 0.00 | 19 | 2.30 | 0.10 | 19 | 1.36 | 0.14 | 10 | 15.88 | 0.00 | 10 | 11.84 | 0.00 |
| 6 | 15 | 152.81 | 0.00 | 27 | 28.41 | 0.00 | 27 | 5.87 | 0.01 | 14 | 12.01 | 0.00 | 14 | 44.33 | 0.00 |
| 7 | 15 | 153.47 | 0.00 | 27 | 12.56 | 0.00 | **27** | **0.00** | **0.27** | 14 | 13.50 | 0.00 | 14 | 47.99 | 0.00 |
| 8 | 16 | 14.40 | 0.00 | 29 | 14.65 | 0.00 | 29 | 2.23 | 0.09 | **15** | **0.00** | **0.42** | **15** | **0.00** | **0.26** |
| 9 | 11 | 284.56 | 0.00 | 19 | 2.63 | 0.09 | 19 | 10.18 | 0.00 | 10 | 29.00 | 0.00 | 10 | 56.40 | 0.00 |
| 10 | 12 | 120.90 | 0.00 | 21 | 2.44 | 0.10 | 21 | 3.42 | 0.05 | 11 | 16.62 | 0.00 | 11 | 11.18 | 0.00 |
| 11 | 12 | 102.71 | 0.00 | **21** | **0.00** | **0.33** | 21 | 3.64 | 0.04 | 11 | 17.63 | 0.00 | 11 | 12.31 | 0.00 |
| 12 | 16 | 154.75 | 0.00 | 29 | 13.92 | 0.00 | 29 | 1.71 | 0.11 | 15 | 13.97 | 0.00 | 15 | 46.42 | 0.00 |
| 13 | 17 | 13.84 | 0.00 | 31 | 15.58 | 0.00 | 31 | 4.78 | 0.02 | 16 | 0.93 | 0.27 | 16 | 0.13 | 0.24 |
| 14 | 17 | 2.74 | 0.20 | 31 | 13.17 | 0.00 | 31 | 2.48 | 0.08 | 16 | 1.52 | 0.20 | 16 | 0.07 | 0.25 |
| 15 | 13 | 99.15 | 0.00 | 23 | 0.81 | 0.22 | 23 | 5.49 | 0.02 | 12 | 18.53 | 0.00 | 12 | 11.50 | 0.00 |
| 16 | **18** | **0.00** | **0.80** | 33 | 14.69 | 0.00 | 33 | 4.24 | 0.03 | 17 | 2.66 | 0.11 | 17 | 0.03 | 0.25 |

In bold are the best models (ΔAICc = 0) for each analysis.
K = number of parameters
*w*_i_ = Akaike weight

Table A3. Effect of habitat characteristics on the number of hatchlings of early and late tree swallow settlers in a nest box network in southern Québec, Canada, between 2009 and 2018, according to the second and third best models in terms of AICc (Table A2).

| Explanatory  variable | Estimate (95% confidence interval) | | | | | | | |
| --- | --- | --- | --- | --- | --- | --- | --- | --- |
|  | Early settlers | | | | Late settlers | | | |
|  | 2nd best *w* = 0.22 | | 3rd best *w* = 0.16 | | 2nd best *w* = 0.14 | | 3rd best *w* = 0.11 | |
|  | Conditional | Zero-inflated | Conditional | Zero-inflated | Conditional | Zero-inflated | Conditional | Zero-inflated |
| Forest 100 m |  |  |  |  |  |  | -0.01  (-0.03, 0.01) | 0.11  (-0.06, 0.28) |
| Forest 5 km |  |  |  |  |  |  | **0.05  (0.01, 0.08)** | -0.12  (-0.45, 0.21) |
| Forage crops 5 km |  |  |  |  |  |  | 0.00  (-0.04, 0.03) | -0.20  (-0.5, 0.10) |
| Forest 5 km ×  Forage crops 500 m |  |  |  |  |  |  | -0.02  (-0.06, 0.01) | **0.65  (0.31, 1.00)** |
| Water 10 km |  |  |  |  |  |  | -0.01  (-0.04, 0.02) | -0.14  (-0.45, 0.17) |
| Insects | 0.01  (0.00, 0.03) | 0.02  (-0.20, 0.23) |  |  |  |  | 0.00  (-0.02, 0.03) | 0.16  (-0.04, 0.35) |
| Competitor density | -0.01  (-0.03, 0.01) | **0.22  (0.02, 0.41)** | **-0.02  (-0.04, -0.01)** | **0.32  (0.17, 0.48)** |  |  | -0.01  (-0.03, 0.01) | **0.25  (0.07, 0.43)** |
| Density @ t-1 | **0.02  (0.00, 0.04)** | -0.15  (-0.35, 0.05) |  |  | **0.02  (0.00, 0.04)** | -0.10  (-0.28, 0.08) |  |  |
| Success @ t-1 | 0.00  (-0.02, 0.01) | -0.16  (-0.33, 0.02) |  |  | 0.02  (0.00, 0.04) | **-0.20  (-0.39, -0.01)** |  |  |
| Temperature | **-0.02  (-0.03, 0.00)** | 0.23  (-0.10, 0.56) | -0.01  (-0.03, 0.00) | 0.23  (-0.07, 0.52) | -0.02  (-0.05, 0.01) | 0.04  (-0.15, 0.23) | -0.02  (-0.05, 0.02) | -0.08  (-0.28, 0.12) |
| Precipitations | 0.01  (0.00, 0.03) | 0.01  (-0.17, 0.19) | 0.01  (0.00, 0.03) | 0.00  (-0.19, 0.18) | 0.02  (0.00, 0.04) | -0.13  (-0.31, 0.05) | 0.01  (-0.01, 0.03) | -0.17  (-0.35, 0.01) |
| Longitude | 0.01  (-0.01, 0.03) | 0.09  (-0.10, 0.29) | 0.01  (-0.01, 0.03) | 0.09  (-0.10, 0.27) | 0.01  (-0.01, 0.04) | 0.03  (-0.16, 0.21) | -0.01  (-0.05, 0.03) | 0.23  (-0.12, 0.59) |
| Latitude | **-0.02  (-0.04, 0.00)** | 0.04  (-0.13, 0.21) | **-0.02  (-0.04, 0.00)** | 0.05  (-0.12, 0.22) | 0.01  (-0.01, 0.03) | 0.02  (-0.17, 0.21) | **0.03  (0.00, 0.05)** | -0.25  (-0.51, 0.01) |

Predictors were standardized (zero mean, unit variance). The Akaike weight of each model is presented along with the estimates and their 95% confidence intervals. Estimates for which the confidence interval excludes zero are in bold.

| Explanatory  variable | Estimate (95% confidence interval) | | | |
| --- | --- | --- | --- | --- |
|  | Early settlers | | Late settlers | |
|  | 2nd best *w* = 0.27 | 3rd best *w* = 0.20 | 2nd best *w* = 0.25 | 3rd best *w* = 0.25 |
| Forest 100 m | 0.1 (-0.08, 0.28) | 0.10 (-0.07, 0.28) | **-0.48 (-0.70, -0.26)** | **-0.49 (-0.71, -0.27)** |
| Forest 5 km | -0.46 (-0.95, 0.04) | -0.45 (-0.94, 0.04) | 0.07 (-0.35, 0.49) | 0.04 (-0.38, 0.45) |
| Forage crops 5 km | **0.54 (0.27, 0.81)** | **0.53 (0.26, 0.80)** | -0.01 (-0.31, 0.30) | -0.01 (-0.31, 0.29) |
| Forest 5 km ×  Forage crops 5 km | **-0.56 (-0.88, -0.23)** | **-0.59 (-0.91, -0.27)** | -0.19 (-0.51, 0.13) | -0.18 (-0.50, 0.14) |
| Water 10 km | **0.59 (0.15, 1.02)** | **0.56 (0.12, 0.99)** | -0.25 (-0.65, 0.15) | -0.22 (-0.62, 0.18) |
| Insects | 0.08 (-0.07, 0.23) |  | -0.13 (-0.31, 0.04) |  |
| Competitor density |  | -0.06 (-0.23, 0.10) | -0.16 (-0.37, 0.05) | -0.15 (-0.36, 0.06) |
| Density @ t-1 | -0.02 (-0.19, 0.14) | -0.04 (-0.22, 0.13) | **-0.51 (-0.72, -0.31)** | **-0.50 (-0.70, -0.29)** |
| Success @ t-1 | **-0.25 (-0.37, -0.12)** | **-0.25 (-0.38, -0.13)** | **0.37 (0.23, 0.52)** | **0.39 (0.25, 0.54)** |
| Temperature | 0.22 (-0.08, 0.52) | 0.26 (-0.04, 0.55) | -0.10 (-0.57, 0.37) | -0.14 (-0.61, 0.32) |
| Precipitations | 0.04 (-0.07, 0.15) | 0.05 (-0.06, 0.16) | 0.00 (-0.13, 0.13) | -0.01 (-0.14, 0.12) |
| Longitude | -0.21 (-0.70, 0.28) | -0.20 (-0.69, 0.28) | 0.09 (-0.34, 0.52) | 0.06 (-0.37, 0.50) |
| Latitude | 0.21 (-0.15, 0.58) | 0.21 (-0.15, 0.58) | **-0.36 (-0.70, -0.02)** | **-0.39 (-0.72, -0.05)** |

Table A4. Effect of habitat characteristics on the fledging success of early and late tree swallow settlers in a nest box network in southern Québec, Canada, between 2009 and 2018, according to the second and third best models in terms of AICc (Table A2).

Predictors were standardized (zero mean, unit variance). The Akaike weight of each model is presented along with the estimates and their 95% confidence intervals. Estimates for which the confidence interval excludes zero are in bold.

Results using the 500-m scale for forage crops and forest cover.

The same preference patterns were observed when forage crops and forest cover were measured at the 500-m and 5-km scales, the only difference appearing when forest cover reached ≥ 50% of the measured area (Fig. 3 and A4). At the 500-m scale, individuals preferred landscapes where open areas were dominated by row crops, but this pattern did not stand out at 5 km.

Both spatial scales yielded the same results regarding early settlers’ number of hatchlings. In late settlers however, we could not detect any effect of landscape context nor of house sparrow density when measuring forage crops and forest at 500 m. Yet, we found that the number of hatchlings increased with the prior year density of tree swallows on the farm, while the probability of hatching failure decreased with the mean number of fledglings produced on the farm in the previous year (Table A6).

Using the 500-m scale, we could not detect any effect of forage crops, the interaction between forage crops and forest, or water cover on fledging success in early settlers. This would lead to different conclusions on the adaptiveness of these variables as potential cues for habitat selection. However, the effects of conspecific social information were the same at both scales. For late settlers, both spatial scales yielded the same results.

| Table A5. Results of the model selections made on the candidate models presented in Table A1 for the different response variables: nest box preference, number of hatchlings and fledging success of tree swallows in southern Québec, Canada, between 2009 and 2018, using a 500-m rather than 5-km scale for forage crops and forest cover. | | | | | | | | | | | | | | | |
| --- | --- | --- | --- | --- | --- | --- | --- | --- | --- | --- | --- | --- | --- | --- | --- |
| Candidate model | Preference | | | Number of hatchlings | | | | | | Fledging success | | | | | |
|  |  |  |  | Early settlers | | | Late settlers | | | Early settlers | | | Late settlers | | |
|  | K | ΔAICc | *w* | K | ΔAICc | *w* | K | ΔAICc | *w* | K | ΔAICc | *w* | K | ΔAICc | *w* |
| 0 | 5 | 288.95 | 0.00 | 7 | 23.34 | 0.00 | 7 | 1.13 | 0.26 | 4 | 7.14 | 0.01 | 4 | 55.86 | 0.00 |
| 1 | 9 | 280.91 | 0.00 | 15 | 19.24 | 0.00 | 15 | 7.94 | 0.01 | 8 | 10.69 | 0.00 | 8 | 58.51 | 0.00 |
| 2 | 14 | 168.94 | 0.00 | 25 | 33.50 | 0.00 | 25 | 15.21 | 0.00 | 13 | 15.04 | 0.00 | 13 | 50.17 | 0.00 |
| 3 | 10 | 282.33 | 0.00 | 17 | 19.32 | 0.00 | 17 | 10.69 | 0.00 | 9 | 11.08 | 0.00 | 9 | 55.57 | 0.00 |
| 4 | 10 | 280.73 | 0.00 | 17 | 2.88 | 0.08 | 17 | 7.74 | 0.01 | 9 | 12.71 | 0.00 | 9 | 60.56 | 0.00 |
| 5 | 11 | 121.05 | 0.00 | 19 | 1.24 | 0.18 | **19** | **0.00** | **0.45** | **10** | **0.00** | **0.36** | 10 | 13.08 | 0.00 |
| 6 | 15 | 170.05 | 0.00 | 27 | 33.90 | 0.00 | 27 | 18.85 | 0.00 | 14 | 15.31 | 0.00 | 14 | 49.48 | 0.00 |
| 7 | 15 | 170.11 | 0.00 | 27 | 17.44 | 0.00 | 27 | 15.20 | 0.00 | 14 | 17.02 | 0.00 | 14 | 52.26 | 0.00 |
| 8 | 16 | 15.91 | 0.00 | 29 | 16.26 | 0.00 | 29 | 10.88 | 0.00 | 15 | 3.20 | 0.07 | **15** | **0.00** | **0.31** |
| 9 | 11 | 282.11 | 0.00 | 19 | 4.32 | 0.04 | 19 | 10.40 | 0.00 | 10 | 13.04 | 0.00 | 10 | 57.63 | 0.00 |
| 10 | 12 | 120.14 | 0.00 | 21 | 1.39 | 0.16 | 21 | 2.06 | 0.16 | 11 | 0.74 | 0.25 | 11 | 12.41 | 0.00 |
| 11 | 12 | 103.90 | 0.00 | **21** | **0.00** | **0.33** | 21 | 3.42 | 0.08 | 11 | 2.03 | 0.13 | 11 | 14.09 | 0.00 |
| 12 | 16 | 171.11 | 0.00 | 29 | 19.29 | 0.00 | 29 | 18.75 | 0.00 | 15 | 17.15 | 0.00 | 15 | 51.57 | 0.00 |
| 13 | 17 | 14.17 | 0.00 | 31 | 16.60 | 0.00 | 31 | 14.11 | 0.00 | 16 | 3.87 | 0.05 | 16 | 0.72 | 0.22 |
| 14 | 17 | 4.05 | 0.12 | 31 | 15.48 | 0.00 | 31 | 13.82 | 0.00 | 16 | 5.24 | 0.03 | 16 | 0.32 | 0.27 |
| 15 | 13 | 100.43 | 0.00 | 23 | 0.85 | 0.22 | 23 | 5.38 | 0.03 | 12 | 2.79 | 0.09 | 12 | 13.28 | 0.00 |
| 16 | **18** | **0.00** | **0.88** | 33 | 16.56 | 0.00 | 33 | 16.96 | 0.00 | 17 | 5.83 | 0.02 | 17 | 0.89 | 0.20 |

In bold are the best models (ΔAICc = 0) for each analysis.
K = number of parameters
*w* = Akaike weight

| Explanatory variables | Estimates (95% confidence intervals) | | | | | | |
| --- | --- | --- | --- | --- | --- | --- | --- |
|  | Preference *N* = 2915 | Number of hatchlings | | | | Fledging success | |
|  |  | Early settlers *N* = 1268 | | Late settlers *N* = 891 | | Early settlers *N* = 953 | Late settlers *N* = 644 |
|  |  | Conditional | Zero-inflated | Conditional | Zero-inflated |  |  |
| Forest 100 m | **-0.60  (-0.73, -0.48)** |  |  |  |  |  | **-0.45  (-0.69, -0.21)** |
| Forest 500 m | 0.03  (-0.18, 0.24) |  |  |  |  |  | -0.25  (-0.58, 0.08) |
| Forage crops 500 m | -0.03  (-0.22, 0.16) |  |  |  |  |  | -0.16  (-0.47, 0.16) |
| Forest 500 m × Forage crops 500 m | **-0.30  (-0.49, -0.11)** |  |  |  |  |  | -0.15  (-0.46, 0.16) |
| Water 10 km | -0.04  (-0.30, 0.21) |  |  |  |  |  | -0.11 (-0.51, 0.29) |
| Insect biomass | **0.13  (0.02, 0.24)** |  |  |  |  |  |  |
| Competitor density | **0.25  (0.13, 0.36)** | -0.01  (-0.03, 0.01) | **0.20  (0.01, 0.39)** |  |  |  |  |
| Density @ t-1 | **0.69  (0.56, 0.82)** | **0.02  (0.00, 0.04)** | -0.16  (-0.36, 0.03) | **0.02  (0.00, 0.04)** | -0.10  (-0.28, 0.08) | -0.08  (-0.24, 0.09) | **-0.44  (-0.62, -0.25)** |
| Success @ t-1 | **0.40  (0.31, 0.50)** | -0.01  (-0.02, 0.01) | -0.16  (-0.33, 0.01) | 0.02  (0.00, 0.04) | **-0.20  (-0.39, -0.01)** | **-0.24  (-0.36, -0.11)** | **0.42  (0.28, 0.57)** |
| Temperature | 0.00  (-0.13, 0.12) | -0.01  (-0.03, 0.00) | 0.24  (-0.07, 0.56) | -0.02  (-0.05, 0.01) | 0.04  (-0.15, 0.23) | 0.26  (-0.05, 0.58) | -0.17  (-0.65, 0.30) |
| Precipitations | **-0.14  (-0.23, -0.05)** | 0.01  (0.00, 0.03) | 0.01  (-0.17, 0.19) | 0.02  (0.00, 0.04) | -0.13  (-0.31, 0.05) | 0.08  (-0.03, 0.19) | -0.02  (-0.15, 0.11) |
| Longitude | -0.13  (-0.38, 0.12) | 0.01  (-0.01, 0.03) | 0.09  (-0.09, 0.28) | 0.01  (-0.01, 0.04) | 0.03  (-0.16, 0.21) | 0.23  (-0.10, 0.56) | 0.18  (-0.19, 0.56) |
| Latitude | -0.15  (-0.35, 0.04) | **-0.02  (-0.04, 0.00)** | 0.03  (-0.14, 0.20) | 0.01  (-0.01, 0.03) | 0.02  (-0.17, 0.21) | -0.17  (-0.48, 0.14) | **-0.48  (-0.79, -0.18)** |

Table A6. Determinants of nest box preference in tree swallows and their effect on the reproductive success of early and late settlers in a nest box network in southern Québec, Canada, between 2009 and 2018, using a 500-m rather than 5-km scale for forage crops and forest cover.

Coefficients come from an ordinal logistic mixed regression for preference, a zero-inflated GLMM using generalized Poisson distribution and log link function for the number of hatchlings and GLMM using binomial error distribution and logit link function for fledging success. Predictors were standardized (zero mean, unit variance). For each analysis we present the coefficients of the model that ranked best in terms of AICc, their 95% confidence intervals and the sample size. See Table 1 for definitions the variables and Table A5 for results of the model selection. Estimates for which the confidence interval excludes zero are in bold.
