# Supplementary material for: Nonideal nest box selection by tree swallows breeding in farmlands: Evidence for an ecological trap?
Source: Ecol Evol. 2021 Nov 9;11(22):16296–313. doi: 10.1002/ece3.8323 (PMC8601888; doi:10.1002/ece3.8323)
Supplement: Supplementary file 6 — Supplementary Material [file ECE3-11-16296-s003.docx]

Figure A1. Results of the model selection by AICc aiming to identify key spatial scale at which the cover of a) forest, b) forage crops, and c) water bodies and wetlands mostly affects nest box preference in a tree swallow population breeding in farmlands in southern Québec, Canada, between 2009 and 2018.

Figure A2. Correlation matrix of the explanatory variables used to assess the determinants of habitat preference and their impact on the reproductive success of tree swallows in a nest box network in southern Québec, Canada, between 2009 and 2018. See Table 1 for the description and justification of the variables.

Figure A3. Averaged predicted fledging success of early and late settlers as a function of mean performance of breeders on a farm in the prior year, for tree swallows in a nest box network in southern Québec, Canada, between 2009 and 2018. Multimodel inference was made on the list of models presented in Table A1; see Table A2 for the Akaike weights. Other variables in the model were kept at their average value. The points represent raw data, and shaded areas are 95% confidence intervals. Blue = early settlers, *N*=953. Green = late settlers, *N*= 644.

Figure A4. Effect of agricultural intensity and openness of landscape within 500 m around nest box on nest box preference for tree swallows in a gradient of agricultural intensification in southern Québec, Canada, between 2009 and 2018. Preference predictions were based on an ordinal mixed logistic regression (model #16 in Table A1). See Table A5 for details on model selection. Other variables in the model were kept at their average value. Shaded areas represent 95% confidence intervals. *N* = 2915 potential breeding attempts along 10 years on 40 farms.
